# Heterogeneity of Alzheimer’s disease: consequence for drug trials?

**DOI:** 10.1186/s13195-018-0455-y

**Published:** 2018-12-19

**Authors:** Gayatri Devi, Philip Scheltens

**Affiliations:** 1 SUNY Downstate Medical Center, Attending Physician, Lenox Hill Hospital | Northwell Health, 65 East 76th St, New York, NY 10021 USA; 2 VU University Medical Center, Alzheimer’s Center of VU University Medical Center, Amsterdam, the Netherlands

**Keywords:** Alzheimer’s, Clinical trials, Heterogeneity, Precision medicine

## Abstract

**Background:**

Alzheimer’s disease is a heterogenous disorder with multiple phenotypes and genotypes, although they eventually converge to a final common clinicopathological endpoint. However, Alzheimer’s disease drug trials do not account for the heterogeneity of the disease in trial design, impeding development of effective drugs.

**Discussion:**

Alzheimer’s disease drug trials commonly have wide inclusion criteria that subsume multiple subtypes of the condition, with varying genotypes, phenotypes, and clinical courses. The outcome variables used in many trials may not be sensitive for the particular disease subtype and trials may not follow patients for the appropriate length of time necessary for the subtype of disease. Methods of stratifying treatment trial design to account for disease heterogeneity using algorithms incorporating demographics, neuroimaging, genetics, and clinical phenotypes, as well as more tailored outcome measures, are proposed to allow for personalized, precision medicine in Alzheimer’s disease therapeutics development.

**Summary:**

Approaching Alzheimer’s disease as a heterogenous disorder will likely improve yield in the search for effective treatments for the condition.

## Background

The current landscape of Alzheimer’s disease drug trials is gloomy. One possible reason for this is that trials are designed as though Alzheimer’s were a monolithic disease. Yet, Alzheimer’s disease includes several subtypes with different clinical phenotypes and degrees of pathology, associated with varying demographics and clinical courses [1–5]. It is important to reliably quantify this heterogeneity to determine the efficacy of any drug [6].

Consider Ms. Y, a 65-year-old clerical worker with aphasia and memory loss beginning at age 55, forcing her to retire at age 58. Diagnosed with Alzheimer’s disease at age 60, she progressed over the next 5 years, with her mini-mental state examination (MMSE) score declining to 18 at age 65, with increasing difficulty functioning.

She then enrolled in a treatment trial, satisfying the inclusion criteria of being between 50 and 88 years of age, scoring between 16 and 26 on the MMSE, and with laboratory and neuroimaging data consistent with probable Alzheimer’s disease. However, these common criteria encompass vast phenotypic and genotypic variability.

Mr. Z, a 77-year-old financier, was eligible for the same trial although his Alzheimer’s disease was quite different from Ms. Y’s. He denied issues but was evaluated at his wife’s insistence for two years of cognitive decline. Diagnosed at age 76, with a positive amyloid scan, his MMSE was 25 a year later. He continued to oversee his company’s operations without significant language disturbances.

These two patients illustrate the heterogeneity of Alzheimer’s disease, biomarker proven, but with widely disparate age at onset, clinical presentation, and course. Yet, in the trial, they would be grouped together, and followed for about a year and a half using various outcome measures, including the MMSE and the Alzheimer’s Disease Assessment Scale-Cognitive (ADAS-Cog) subscale scores. However, one would expect them to have different outcomes based on the variability at entry and the outcome measures chosen. Any efficacy of the agent would be ameliorated by the heterogeneity of the group—based on genetics, co-morbidities, brain regions affected, and preexisting cognitive and brain reserves [6].

## Discussion

Current Alzheimer’s disease treatment trial design is associated with several concerns. The age range for inclusion in Alzheimer’s trials encompasses both early- and late-onset Alzheimer’s patients. People with sporadic, early-onset Alzheimer’s disease like Ms. Y generally have more aggressive courses than those with late-onset Alzheimer’s disease like Mr. Z. Controlling for age does not account for inherent phenotypic differences between early and late-onset Alzheimer’s disease. Even within early-onset Alzheimer’s disease, significant variability in phenotype, pathology, and disease duration exists, modified by mutations and variables such as apolipoprotein E(APOE) status [7]. Aside from the age at onset difference, Ms. Y and Mr. Z had different subtypes, with Ms. Y’s form, with language impairment, being more aggressive [4]. Various subtypes of late-onset Alzheimer’s disease have been described, with distinct demographics, clinical phenotypes, and neuroimaging and cognitive profiles. In a cohort of 648 patients with repeated follow-up, the mean length of commonly used Alzheimer’s disease stages was comparable to their standard deviations, suggesting a high level of interindividual variability, which may make drug efficacy difficult to establish [6].

In a well-delineated cohort of 938 patients, eight different subtypes of Alzheimer’s disease based on neurocognitive measures were found [5]. In a cluster analysis of four large cohorts, patients sorted into memory impaired and memory spared clusters, the latter being younger with more aggressive disease [8]. An anatomical study of regional atrophy patterns with neuroimaging found four different subtypes of disease [4]. In an autopsy series of 889 patients with Alzheimer’s disease, neurofibrillary tangle location distinguished three types with dramatically different rates of deterioration, from five points annually on the MMSE to just a point a year [3]. Another study found five different subgroups of sporadic Alzheimer’s disease with different clinical profiles based on CSF levels of AB1–42, tau, and ubiquitin [9]. The advent of tau neuroimaging has led to the realization that patients with Alzheimer’s disease who have similar amyloid loads may differ clinically based on the location of tau deposits [10]. These studies speak to the significant biological variation in Alzheimer’s disease.

Coexisting systemic and brain illnesses further influence clinical course. Systemic conditions such as cardiac disease and diabetes, brain pathology such as Lewy body disease, and ischemic brain disease are common. While treatment trials exclude those patients with poorly controlled systemic illnesses or those who have had large strokes, the concomitant presence of milder or well controlled versions of these illnesses has disease-modifying effects. Lewy body disease, a common comorbidity, affects clinical presentation and age of onset [11]. Argyrophilic grain disease, a tauopathy with memory impairment found in 30% of patients with dementia, is almost never diagnosed clinically and often missed at autopsy [12]. How these commonly coexisting, antemortem-undetected conditions modify the course of biomarker-proven Alzheimer’s disease is unknown.

The length of most Alzheimer’s disease drug trials may be insufficient to estimate the effect of intervention for some subtypes. The time needed to observe efficacy of a drug in the aggressive, hippocampal-sparing Alzheimer’s disease subtype might be shorter than that needed for the more benign limbic-predominant subtype. Among cognitively normal individuals aged 70 and older, just 4 of 88 with Alzheimer’s pathology developed symptoms, even after 2.5 years of follow-up, underscoring the need for adequate length of monitoring in trials [13] . Furthermore, equivalent brain pathology more often presents with different severity and clinical symptoms as a function of each person’s unique preexisting brain resilience and cognitive reserve. Some disease-modifying medications have advanced to phase 3 trials without dose optimization or clear evidence of target engagement, sometimes with retrospective analysis of responders to design the phase 3 trials, negating randomization. Finally, choosing the appropriate outcome variable for the disease subtype being evaluated is also important.

We propose that, aside from using biomarkers to define the presence of Alzheimer’s disease, drug trials in non-familial cases should incorporate finer definitions of neurocognitive and neuroimaging parameters, genetic status, demographics, and co-morbidities that affect the course in phase 2 trials. Patients would be a priori stratified into subgroups by performing cluster-based analysis in existing large datasets, defining relatively homogenous patient groups where precision medicine can be implemented to guide therapeutics. Appropriate outcome variables, sensitive to the subgroup, should be used. An algorithm derived from B-amyloid and tau scans, fluorodeoxyglucose PET, and anatomic, blood flow, and resting phase MRI generated an index of gene expression that was superior to the MMSE and ADAS-Cog in predicting treatment response [14]. Delineating such patient clusters also allows for treatment targeting specific deficits, such as language. Additional stratification, either by known mutations in early-onset patients or using a polygenic hazard ratio in late-onset patients, obtained using disease-associated single nucleotide polymorphisms and APOE status, may further refine populations for therapeutic trials [15].

Phase 3 trials would follow these groups individually, rather than drawing conclusions from untenably broad definitions of Alzheimer’s disease. While we acknowledge that such methods involve more expense to recruit sufficient numbers of patients with longer follow-up periods, we believe this to be a more productive approach. Another strategy would focus solely on neuropathologic load reduction, as all patients, regardless of clinical phenotype or genetic mutation, manifest the same pathology. However, inconsistent correlation exists between neuropathology and symptom onset, particularly in older individuals, with 41% of cognitively normal persons, aged 80–89, having positive amyloid scans [16].

## Summary

The one-treatment-fits-all approach applies even less to Alzheimer’s disease than it does to other common, chronic, and complex illnesses. In approaching treatment trials, the question to ask is what are the particulars of the individual’s particular Alzheimer’s? Subsumed under the Alzheimer’s disease label are numerous subtypes—additionally influenced by coexisting brain and systemic pathologies—defined by a combination of genetics, comorbidities, anatomy, and clinical phenotypes, with different responses to treatment and different prognoses, all leading to the same, final clinicopathological endpoint. Therapeutic trial designs incorporating this approach may improve yield in Alzheimer’s disease.
